# The Potential of Colonic Tumor Tissue *Fusobacterium nucleatum* to Predict Staging and Its Interplay with Oral Abundance in Colon Cancer Patients

**DOI:** 10.3390/cancers13051032

**Published:** 2021-03-01

**Authors:** Pamela Pignatelli, Lorena Iezzi, Martina Pennese, Paolo Raimondi, Anna Cichella, Danilo Bondi, Rossella Grande, Roberto Cotellese, Nicola Di Bartolomeo, Paolo Innocenti, Adriano Piattelli, Maria Cristina Curia

**Affiliations:** 1 Department of Medical, Oral and Biotechnological Sciences, “G. d’Annunzio” University of Chieti-Pescara, Via dei Vestini, 66100 Chieti, Italy; pamelapignatelli89p@gmail.com (P.P.); lorenaie@outlook.com (L.I.); martyy96@hotmail.it (M.P.); roberto.cotellese@unich.it (R.C.); apiattelli@unich.it (A.P.); 2 Department of General Surgery, Private Hospital “Villa Serena”, Città Sant’Angelo, 65013 Pescara, Italy; paolo.rai@gmail.com (P.R.); annacichella@libero.it (A.C.); ncldibartolomeo@gmail.com (N.D.B.); paolo.innocenti@yahoo.it (P.I.); 3 Department of Neuroscience, Imaging and Clinical Sciences, “G. d’Annunzio” University of Chieti-Pescara, Via dei Vestini, 66100 Chieti, Italy; danilo.bondi@unich.it; 4 Department of Pharmacy, “G. d’Annunzio” University of Chieti-Pescara, Via dei Vestini, 66100 Chieti, Italy; rossella.grande@unich.it; 5 Villa Serena Foundation for Research, Città Sant’Angelo, 65013 Pescara, Italy

**Keywords:** oral microbiota, *Fusobacterium nucleatum*, colon cancer, abundance, dysbiosis, diet, lifestyle, tumor microenvironment, staging, *Porphyromonas gingivalis*

## Abstract

**Simple Summary:**

Colon cancer (CC) is a multifactorial disease, and complex interactions among the gut microbiota, inflammation and environmental exposures are needed for colorectal carcinogenesis. Oral *Fusobacterium nucleatum* (*Fn*) and *Porphyromonas gingivalis* (*Pg*) are predominant pathogens involved in periodontitis and can migrate from the oral cavity to other districts of the body, including the colon. The aim of the study was to analyze the link between oral *Fn* and *Pg*, oral health, diet, lifestyle and risk of CC. The *Fn* quantity was greater in the oral cavity than in CC, and its concentration influenced the *Fn* quantity in CC tissue. The meat consumption was related to intestinal *Fn*. Instead, *Pg* was not associated with CC. The *Fn* abundance in CC tissue could predict cancer staging, becoming a potential biomarker to find out the prognosis of colic cancer patients.

**Abstract:**

Background. Intestinal microbiota dysbiosis may enhance the carcinogenicity of colon cancer (CC) by the proliferation and differentiation of epithelial cells. Oral *Fusobacterium nucleatum* (*Fn*) and *Porphyromonas gingivalis* (*Pg*) have the ability to invade the gut epithelium, promoting tumor progression. The aim of the study was to assess whether the abundance of these odontopathogenic bacteria was associated with colon cancer. We also investigated how lifestyle factors could influence the oral *Fn* and *Pg* abundance and CC. Methods. Thirty-six CC patients were included in the study to assess the *Pg* and *Fn* oral and colon tissue abundance by qPCR. Oral health data, food habits and lifestyles were also recorded. Results. Patients had a greater quantity of *Fn* in the oral cavity than matched CC and adjacent non-neoplastic mucosa (adj t) tissues (*p* = 0.004 and *p* < 0.001). Instead, *Pg* was not significantly detected in colonic tissues. There was an association between the *Fn* quantity in the oral and CC tissue and a statistically significant relation between the *Fn* abundance in adenocarcinoma (ADK) and staging (*p* = 0.016). The statistical analysis revealed a tendency towards a greater *Fn* quantity in CC (*p* = 0.073, η^2^*_p_* = 0.12) for high-meat consumers. Conclusion. In our study, *Pg* was absent in colon tissues but was correlated with the oral inflammation gingival and plaque indices. For the first time, there was evidence that the *Fn* oral concentration can influence colon tissue concentrations and predict CC prognosis.

## 1. Introduction

Increasing evidence has supported that many systemic diseases are associated with disturbances in the oral ecosystem—for example diabetes, cardiovascular diseases and tumors. The most prevalent of these are periodontal diseases and are defined as microbiota-associated diseases. Gingivitis, a reversible inflammatory disease caused by a resident bacterial plaque microbiota, is the most common and prevalent form of periodontal disease. If left untreated, it progresses into periodontitis, a chronic, irreversible inflammatory disease in which alterations in the composition of the microbial community lead to a change in the host–microbe crosstalk. The predominant pathogens involved in periodontitis are *Porphyromonas gingivalis* (*Pg*), *Prevotella intermedia*, *Fusobacterium nucleatum* (*Fn*), *Tannerella forsythia*, *Eikenella corredens*, and *Treponema denticola*.

*Fn*, a Gram-negative anaerobe bacterium, is a common member of the oral microbiota [1,2] linked to plaque biofilm formation and periodontitis [3]. It is considered as an intermediate colonizer bridging the attachment of commensals that colonize the tooth and epithelial surface with true pathogens [4]. *Fn* encodes for adhesin FadA, a best-characterized virulence factor expressed on its bacterial surface, and binds to the cell junction molecules cadherins. In particular, FadA binds to vascular endothelial cadherin (VE-cadherin) on the endothelial cells and to E-cadherin on epithelial cells [5]. *Fn* can also secrete serine proteases and cause the depletion of immune cells at the site of the infection due to the induction of apoptotic cell death. This immunosuppression can lead to the recruitment and the binding of other pathogenic microorganisms [6]. *Pg* is a Gram-negative anaerobic bacterium, periodontal pathogen and associated with periodontitis [7]. This microorganism induces dysbiosis by impairing innate host defenses and can block phagocytosis and promote inflammation, activating the PI3K signaling pathway [8]. Intracellular *Pg* can inhibit apoptosis in gingival epithelial cells by upregulation of the antiapoptotic molecule Bcl-2 [9]. *Pg* produces the cysteine proteinases gingipains, which can cleave the MMP-9 proenzyme and, finally, modulates reactive oxygen species (ROS), mediating, in this way, the activation of the transcription factors associated with inflammation and cancer development [10].

In the last two decades, significant evidence has emerged implicating bacteria in the etiology of some cancer types, such as *Helicobacter pylori* in gastric cancer and mucosa-associated lymphoid tissue (MALT) lymphomas, Chlamydia trachomatis in cervical cancer and Salmonella typhi in gallbladder cancer [11]. *Fn* has been detected in several tumors, including colorectal [12,13], esophageal [14] and pancreatic cancer tissues [15]. *Pg* was detected in oral squamous cell carcinoma (OSCC) [16,17], in esophageal squamous carcinoma (ESCC) [18], in the precancerous lesions of gastric cancer in individuals with periodontal disease [19] and in pancreatic cancer [20].

Among the different mechanisms proposed, the activation of the Wnt/β-catenin and NF-κB oncogenic signaling pathways in epithelial cells represents a well-elucidated mechanism of carcinogenesis [21].

In a healthy gut, the bacteria maintain homeostasis with the host, but, when bacterial dysbiosis occurs, the host may reveal inflammation and a loss of barrier function. Commensal bacteria are effective for the function of the intestinal immune system, being necessary for the full development of Peyer’s patches [22]. Complex interactions among the gut microbiota, inflammation, environmental exposures and host genetics are required for colorectal carcinogenesis.

No single bacterial species has been identified as a risk factor for colorectal cancer (CRC), but some studies have reported an increase of *Fn* abundance in human colorectal tumors compared to controls, and a significant positive correlation between a high *Fn* abundance and the presence of colorectal adenomas has been found [12,23,24]. The adhesin FadA has been identified as a putative bacterial oncogenic driver of CRC. The attachment and invasion take place via adhesin FadA, and it is essential for the induction of inflammation. The FadA gene is unique to *Fn* and, hence, is an ideal potential diagnostic marker. It could bind to the transmembrane or the cytoplasmic domains of E-cadherin on colon cancer cells, activating Wnt/β-catenin signaling and leading to enhanced transcriptional activity and the activation of proinflammatory cytokines, ultimately affecting the development of cancer [25,26,27]. A polymicrobial synergy and dysbiosis (PSD) model has been suggested in which *Fn* can bind epithelial cell by FadA adhesin. *Fn,* described as a “driver” species, promotes epithelial DNA damage and initial tumorigenesis. It can create an altered niche in the colon for colonization by “passenger” aggressive bacteria, such as *Pg*, which may produce virulence factors continuing to transform the biofilm community structure [28]. *Fn*, at first, generates a proinflammatory microenvironment recruiting T-lymphocyte-infiltrating tumors (TIL); then, it promotes carcinogenicity by suppressing the adaptive immunity downregulating the antitumor-mediated immune response by T lymphocytes (natural killer and cytotoxic) and by creating a tumor microenvironment [29,30].

Environmental factors such as smoking and obesity have been shown to be associated with the subgingival and intestinal dysbiotic communities, respectively [31,32,33,34]. Smokers are known to harbor a more pathogenic anaerobic subgingival microbe population than nonsmokers. Indeed, the *Fn* abundance is affected by smoking in both periodontally healthy and diseased individuals [35,36]. A higher severity of the periodontitis and plaque index (PII) has been reported among alcohol users with incremental consumption [37]. Alcohol-dependent users with periodontitis present a worse periodontal status and higher frequency of some periodontal pathogens, such as *Fn* [38]. An excessive intake of alcohol can contribute to an increase in the rate of biofilm formation and accumulation and may affect the host response to infections caused by bacteria, thus increasing the host vulnerability, since alcohol can cause oral dehydration and reduction in salivary flow. An inflammatory diet, red meat, processed meat, refined grain, and sugary beverages were associated with a higher risk of proximal *Fn*-positive colorectal tumors but not with the risk of *Fn*-negative cancers [39]. On the contrary, a diet rich in fibers can decrease the risk of colorectal cancer, as it may influence the bacterial fermentation of foods, changes in pH and the increase in transit time of intestinal contents, creating colonic niches less hospitable for nonlocal species such as *Fn* [40].

The aim of this study is to assess whether the abundance of these odontopathogenic bacteria is associated with colon cancer. This explorative study is the first one investigating the presence of *Fn* and *Pg* both in the oral cavity and in normal and tumor colon tissues and also correlating these results with different risk factors through dedicated questionnaires. Furthermore, the abundance of periodontal bacteria was associated with the plaque and gingival index for the first time in CC patients.

## 2. Results

We investigated the presence of *Fn* and *Pg* in 36 colon cancer patients by quantitative real-time PCR (qPCR). The clinical and histopathological characteristics of patients are shown in Table 1, and the study subject characteristics (information from the questionnaires) are shown in Table 2.

The presence of *Fn* was detected in both the oral cavity and matched cancer tissue and adjacent non-neoplastic mucosa. *Pg* was present in the oral cavity but absent in a representative series of colon tissues.

Within the study cohort, the patients had an average age of 67.17 ± 12.42. Among the colon adenocarcinoma (ADK) patients, the mean number of teeth was 21.05 ± 9.1, and there were two edentulous. The mean PII was 1.4 ± 0.73, and the GI was 0.66 ± 0.67, which indicate nonoptimal oral hygiene with abundant plaque. Where it was possible, dietary data and patients’ smoking habits were recorded. Most of the patients were nondrinkers/light drinkers and nonsmokers (Table 2).

### 2.1. Quantitative Molecular Analysis

**Figure 1:**
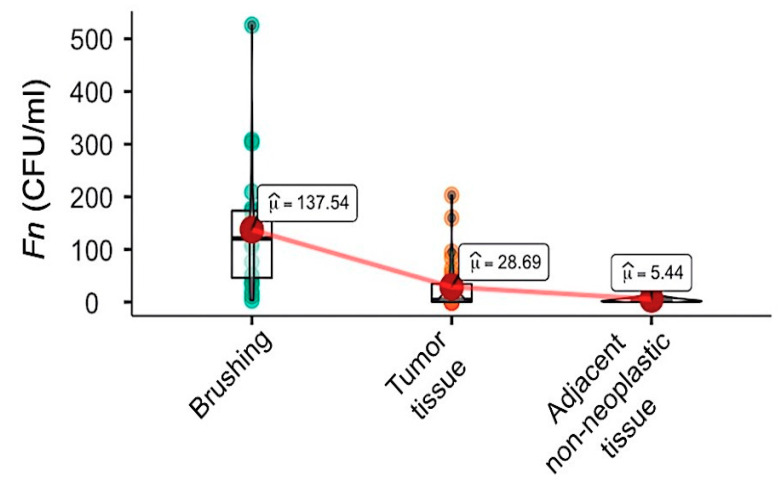
*Fusobacterium nucleatum* (*Fn*) abundances in matched oral/colonic tissues from colon cancer patients by qPCR. The figure shows the violin plots of *Fn* in the two body districts, oral cavity and colon tissues (tumor tissue and adjacent non-neoplastic colon mucosa) of the cases examined. Big dots represent 10% trimmed mean (μ), and related box plots represent median and interquartile range. CFU: colony-forming units.

The quantitative molecular analysis revealed that oral *Fn* was more abundant in the oral cavity (*Median(Mdn)* = 108.69 colony-forming units (CFU)/mL) than cancer tissue (*Mdn* = 4.78 CFU/mL) (Figure 1 and Supplementary Table S1) (Wilcoxon test: *p* = 0.004, robust test: *p* = 0.059, dunbiased (dunb) = 0 0.332) and adjacent tissue (*Mdn* = 2.19 CFU/mL) (Wilcoxon test: *p* < 0.001; robust test: *p* = 0.038, dunb = 0.382). Though not significantly, the *Fn* quantities were greater in colon cancer rather than in the adjacent non-neoplastic tissue (Wilcoxon test: *p* = 0.100, robust test: *p* = 0.066, dunb = 0.253) (Supplementary Table S2).

There were no differences in the *Fn* quantity in colon cancer tissue nor in the adjacent one, depending on the cancer localization (ascending, transverse, descending or sigmoid colon). Male and female patients did not differ in the quantity of *Fn*, both in tumoral and adjacent non-neoplastic tissue. Yet, no difference was found for oral *Fn* or *Pg* depending on gender. Our study did not detect *Pg* in colonic tissues.

### 2.2. Association Analysis among Bacteria Abundance, Lifestyle Data and Oral Health Status

In the patients, fruit consumption was low for 31.4% of participants and high for 68.6%. Vegetable consumption was low for 71.4% and high for 28.6%. Meat consumption was low for 34% and high for 66%. Fifty percent of the patients frequently consumed meat cooked at high temperatures (fried or barbecued) (Supplementary Figure S1). There was a low percentage (12.5%) of high alcoholic consumption among the participants, and 25.7% of them consumed no alcoholic beverages. Finally, 75% of the participants did not report any current or previous smoking habit, whereas 25% of them currently smoked, and 22% were past smokers.

The number of teeth, though not significantly, differed based on fruit consumption (*p* = 0.053, η^2^*_p_* = 0.32, R^2^_adj_ = 0.23): the post-hoc analysis revealed higher consumers to have a greater number of teeth compared to low consumers (mean difference = 7.75, *p* = 0.046). Neither significance nor other strong tendencies were found for the other lifestyle factors on the oral health indices.

Meat consumption was related to intestinal *Fn*, as the statistical analysis revealed a tendency for high consumers having greater quantities both in the adjacent non-neoplastic tissue (*p* = 0.037, η^2^*_p_* = 0.18, R^2^_adj_ = 0.15) and in the cancer tissue (*p* = 0.050, η^2^*_p_* = 0.14, R^2^_adj_ = 0.11). No other effects were found for the lifestyle factors on the bacteria quantities.

**Figure 2:**
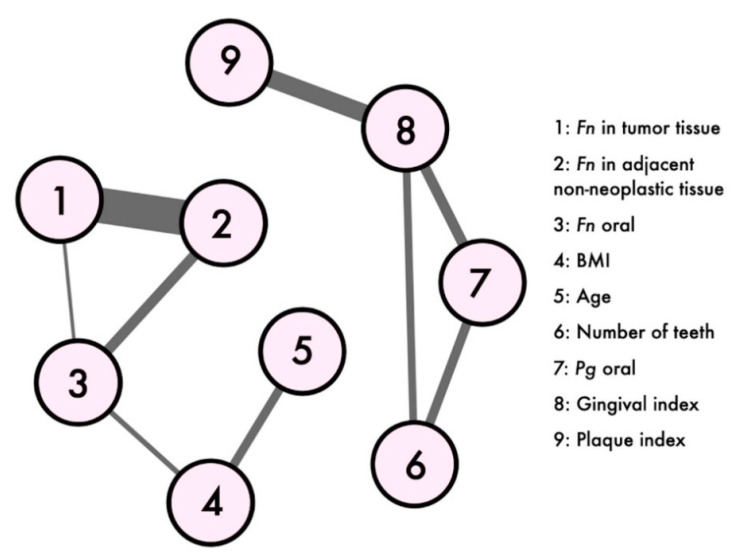
Network plot. Network plot of the cancer patients variables, including the *Fn* and *Porphyromonas gingivalis* (*Pg*) quantities, oral health parameters, age and body mass index (BMI); labels are shown for weights over 0.24 Spearman correlation coefficients (rho). The thicker the line, the highest the weight of the correlation between the nodes. Nodes were positioned using the Fruchterman-Reingold algorithm. Density: 0.25, number of observations: 34 and missed values were excluded pairwise.

The correlation matrix (Table 3) showed significant correlations between the plaque index and gingival index (*p* = 0.020, rho = 0.52). Tendencies were revealed for oral *Pg* compared with the gingival index (*p* = 0.121, rho = 0.38) and number of teeth (*p* = 0.146, rho = 0.36). Other tendencies were revealed comparing the number of teeth and gingival index (*p* = 0.159, rho = −0.33), oral *Fn* and body mass index (BMI) (*p* = 0.162, rho = 0.29). Even though the characteristics of the network analysis herein presented did not allow a robust clustering analysis, the graph (Figure 2) shows that two main clusters emerged: the one constituted by the quantities of *Fn* in the oral and intestinal tissues and the other constituted by the oral health indices. Of note was that *Pg* was part of the second cluster, indicating that the links of this population quantities differed from those of *Fn*. The centrality measures revealed *Fn* in oral tissue to be the main node of the network, achieving the highest values both for eigenvector centrality and betweenness centrality, and the second rank for the closeness centrality, after the gingival index.

**Figure 3:**
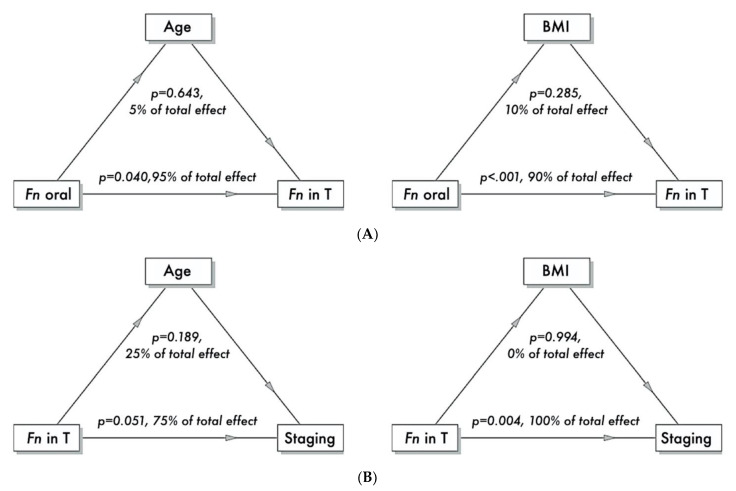
Mediation analysis. Panel (**A**) Quantity of *Fn* in the oral tissue was the predictor, quantity of *Fn* in the tumoral tissue the dependent variable and age or BMI the mediators. The analysis revealed the age and BMI exerted only a trivial mediation (5% and 10%, respectively) on the conditional dependence between *Fn* in the oral and tumoral tissues. Panel (**B**) Quantity of *Fn* in the tumor tissue (T) was the predictor, staging the dependent variable, and age or BMI the mediators. The analysis revealed that age exerted only a small mediation (25%, even though not significantly), whereas the BMI did not exert any, on the conditional dependence between *Fn* in the tumoral tissue and cancer staging.

The *Fn* oral quantity, though not significantly, had a moderate predictive power for the *Fn* quantity in cancer intestinal tissue (*p* = 0.056, r = 0.35, root mean square error (RMSE) = 1.44); the r value corresponds to a Cohen’s d value of 0.75 [41]. Instead, a lower effect, neither significant nor with a statistical tendency, was found for the *Fn* quantity in oral tissue for predicting the *Fn* quantity in the non-neoplastic intestinal tissue (*p* = 0.284, r = 0.21, RMSE = 1.04). The further mediation analysis revealed the mediation of either age or BMI to be trivial (Figure 3A).

### 2.3. Association Analysis between Bacteria Abundance and Staging

The *Fn* quantity in the oral tissue did not predict the staging nor the grading of the colon ADK, whereas the *Fn* quantity in the cancer tissue significantly predicted the staging (*p* = *0*.016) but not the grading (Table 4). A similar tendency was found for the *Fn* quantities in the non-neoplastic intestinal tissue in predicting the staging. The further mediation analysis revealed age to lightly mediate this relation, whereas the mediation of the BMI was absent (Figure 3B).

The network analysis revealed *Fn* in oral brushing to be the main node of the network. Together with the mediation analysis, this study showed how *Fn* present in the oral cavity influenced the presence of the bacterium in apparently normal colon mucosa but, especially, in tumor tissue. In all of this, the mediators such as age and lifestyle had a low effect. In turn, the presence of the bacterium in the tumor tissue had a statistically significant influence on staging.

## 3. Discussion

In this study, we evaluated the role of the oral microbiota and dietary patterns in the onset of colon cancer.

It has been reported in the literature that the intestinal microbiota played a key role in colonic carcinogenesis, notably the peri-odontogenic bacterium *Fn*. In this study, for the first time, oral *Fn* and *Pg* concentrations were analyzed in association with periodontal indices and bacterial concentrations in colon tissue. Molecular analyses were performed to quantify *Fn* and *Pg* in colon cancer patients in order to assess the presence of any imbalances in the oral microbial flora.

Previous studies showed *Fn* overabundance in tumor tissues of colorectal adenoma and carcinoma patients when compared to matched normal tissues [12,13,23,29].

CRC variations in the incidence rates worldwide are attributed to differences in the exposure to environmental factors—in particular, diet and lifestyle (red and processed meat consumption, smoking, alcohol consumption and BMI)—whereas an inverse association exists with the consumption of vegetables [42].

Similar to the literature, in our results, *Fn* was statistically significant higher in pathological tissue compared to the matched adjacent non-neoplastic mucosa. The *Fn* quantity increased significantly in the colon cancer tissue through the major stages of colorectal neoplasia progression in such a way that predicted the staging (*p* = *0*.016). There was also an association, although not statistically significant, between the *Fn* concentration in the adjacent non-neoplastic mucosa and cancer staging. The network plot revealed a correlation between the *Fn* oral and colon tissue bacterial load and, in particular, a very high correlation between the *Fn* quantities in colon cancer and adjacent non-neoplastic mucosa. The abundance of *Fn* in cancer tissue increased with the oral *Fn* concentration.

A network analysis revealed *Fn* in the oral tissue to be the main node of the network. In particular, this node was ranked the highest for eigenvector centrality, a measure that considers a node important if it is linked to other important nodes that are themselves central [43], and the highest rank for betweenness centrality, which ranks the nodes based on the importance of a node in the average pathway between other pairs of nodes [44]; in addition, it achieved the second-highest rank for closeness centrality, which quantifies the relationship of a node to all other nodes. Although the centrality of a node in undirected networks is based on conditional dependence and cannot be confused with causal inference, the centrality measures represent the underlying dynamical system in a meaningful way [43]. Thus, the network analysis herein applied revealed *Fn* in oral tissue to play a central role in the systemic path of colorectal cancer. Barajas-Martinez and colleagues [45] recently proposed a network approach to investigate the loss of metabolic homeostasis. Indeed, network physiology recently emerged as a theoretical framework and a system-wide approach to understand how the integration of physiological systems, each with its own complex structure and mechanisms of regulation, leads to distinct physiologic and pathophysiologic paths [46]. The pilot evidence of our network analysis opens the door for further larger analyses to generate meaningful integrative models of colon cancer pathophysiology. The role of *Fn* as a poor prognostic factor of CRC patients agrees with what has already been reported about the existence of a link between a high *Fn* DNA load in CRC tissue and the shorter survival of patients [47,48,49,50].

This study did not come without limitations. The sample size and the absence of a control group may have limited the inferring of the results. However, it is worth noting that we conducted three samples in different sites, and all analyses were carried out in triplicate; in addition, the definition of a control group for such a study encompasses both a healthy matched group (normal colon tissue) and benign tumor patients. In the present study, the authors provided a comprehensive assessment of the oral health, microbial quantities and lifestyle habits, adding a control tissue (adjacent non-neoplastic mucosa) for comparing with the tumoral one. For the three main findings—an association between the *Fn* quantity in oral and CC tissue, between the *Fn* abundance in ADK and staging and between the *Fn* quantity in CC and meat consumption—we obtained a statistical power (1−β) of 0.54, 0.51 and 0.74, respectively. Using the effect size, we obtained a setting of α = 0.05 and 1−β = 0.8; the required sample sizes for further analyses are 58, 36 and 56 participants, respectively. Therefore, possible further implementations of our findings may take into account a little bit larger sample size, with the possible requirement of control groups, the quantitative current or the retrospective calculation of micro- and macronutrient daily intakes and sequential analyses to identify the time course of the likely migration of bacteria from oral to cancer tissues.

In this study, *Pg* was not detected in the pathological tissue and adjacent non-neoplastic mucosa, confirming the literature data about a noninvolvement of this bacteria in colon cancer. However, when plotting the *Pg* oral quantities into a network, we found a high correlation between oral *Pg* and the gingival and plaque indices and, also, with the number of teeth, confirming the important role of *Pg* in oral inflammation.

For the first time, *Fn* and *Pg* oral and colonic tissue concentrations were analyzed in association with periodontal indices. *Fn* and *Pg* highlighted different correlations, *Fn* with colon (pathologic) tissues and *Pg* with oral inflammation. *Pg*-associated inflammation is related with oral cancer, as reported by various papers [16,17]. *Pg* infection is correlated with chronic periodontitis and with the formation of an immune microenvironment. This interaction will be responsible for cancer development in the oral cavity rather than in most distant sites.

Data from the literature reported an association between smoking and a significant abundance of seven bacterial species, including *Fn*, and between an intense consumption of alcohol with high oral levels of periodontal-pathogenic bacteria in subgingival plaque, including *Fn* in patients with and without chronic smoker periodontitis [36].

Alcohol consumption increased the intestinal permeability by facilitating the bacterial invasion of epithelial cells, promoting the onset of colorectal cancer [51]. In our study, alcohol consumption did not affect the oral and colonic concentrations of *Fn* and *Pg*, nor the increased risk of CRC, and no significant difference in relation to alcohol consumption was observed. Alcohol was not identified as a factor promoting colon cancer, because the sample examined consisted of 87.5% of nondrinkers/light drinkers. There was no relationship between the habit to smoke with the oral concentration of *Fn* and *Pg*, because in our sample, there were 75% nonsmokers. It should be noted that, in the study cohort, there was a low percentage of smokers and alcohol users, so this could influence the results.

Although a reduction in meat intake could be a primary reason for the reduced risk demonstrated in vegetarians, an increase in the consumption of various whole plant foods might also contribute to the reduction. The data reported a correlation between high levels of protein consumption (particularly, animal protein) during the Middle Age and increased risk of cancer and higher mortality [52]. The cases in our study had a consumption of vegetables below the recommendations of the guidelines, and 66% were heavy consumers of meat, 50% cooked at high temperatures.

This study revealed a relationship between meat consumption and intestinal *Fn*—in particular, a tendency for high consumers to have greater *Fn* quantities both in adjacent non-neoplastic tissue (*p* = 0.037) and in cancer tissue (*p* = 0.050). Recent studies have suggested that the levels of *Fn* represent a valuable marker for CRC diagnosis [53,54] and that a proinflammatory diet might be associated with a higher risk of *Fn*-positive colorectal tumors [39]. There is a strong correlation between diet, microbiota and colon cancer development; in fact, our data confirmed that a diet based on red and processed meat has been associated with colorectal carcinogenesis [53], although the metabolic and inflammatory mechanisms involved remain to be elucidated. It is hypothesized that diet components or their metabolic products affect or modulate the gut microbiota function and composition. A microbial dysbiosis might favor the colonization of pathogens or microbial species that, after changing their favorite habitat or microenvironment, such as *Fn* from the oral cavity moving to the intestinal mucosae, become pathogens, promoting oncogenic pathways. As previously demonstrated, the major carcinogenic factors associated with red and processed meat consumption are heme compounds, heterocyclic amines, N-nitroso compounds (NOCs) and undigested proteins. These components can modify the composition of the gut microbiota, thus promoting CRC development. No influence of the habit to cook meat at high temperatures on colon cancer were found in our series of patients, confirming the recently reported data on ultra-processed food (UPF) consumption and different types of cancer, such as prostate, colorectal, overall breast and premenopausal breast cancers [54]. In order to obtain statistically significant numbers, it would be necessary to carry out a study with a higher sample size. No other effects were found for the lifestyle factors on bacteria quantities.

Finally, the number of teeth, though not significantly, differed based on fruit consumption (*p* = 0.053); furthermore, higher consumers had a greater number of teeth compared to low consumers, regardless of age, stressing how the preservation of dental structures could promote a healthier diet.

Neither a significance nor other strong tendencies were found for the other lifestyle factors on the oral health indices.

The diet—in particular, the endogenous formation of N-nitroso compounds due to increased levels of processed meat consumption—is responsible for alkylation DNA damage and, therefore, genetic DNA aberrations in the intestinal environment [55,56]. Together with the environmental factors, they determine the risk of colorectal neoplasia to a degree similar to genetic predisposition, according to the mechanisms of gene–environment interactions [57]. Actually, advanced age (>65 years) was associated with changes in the microbiome contents and, also, an increased risk of colorectal cancer. Those of the microbiota could be a separate pathway that could induce carcinogenesis without necessarily needing the influence of the other two.

Pathogenic events may happen long before the appearance of clinically detectable precancerous lesions, even in an apparently normal epithelium. *Fn* is a driver that promotes invasion by oral microbes into the colonic mucosa by acting as a bridge between early and late colonizers by binding with them through adhesins [28]. *Fn* is able to influence the tumor microenvironment according to different mechanisms: the avoidance of anticancer immune responses and the increase in the production of reactive oxygen species (ROS) in the intracellular environment due to the inflammatory environment generated by *Fn* in the colonic mucosa [58]. High levels of ROS favor oncogenic mutations in DNA and can reduce the enzymatic activity of mismatched repair proteins due to MutL homolog 1 (MLH1) gene silencing, thus favoring microsatellite instability. In addition, ROS facilitate the onset of the methylator phenotype [6]. In colon cells, *Fn* also induces the production of metalloproteases, which play a primary role in inflammation and tumor invasion, and promotes an uncontrolled activation of the Wnt pathway [29]. This study revealed that *Fn* in colon tissue played an important role in the acquisition of the oncogenic phenotype. Thus, *Fn* may be considered as a prognostic biomarker but, also, a therapeutic target of colon cancer.

The study from Komiya et al. reported identical *Fn* strains in the saliva and CRC tissues in the same patient, defining a link between oral and colonic *Fn* and suggesting that colon *Fn* originates from the oral cavity [59]. However, it is not yet clear how *Fn* reaches the colon from the oral cavity. Recently some authors reported that *Fn* may migrate to the colon by descending via the digestive tract or using the circulation system during transient bacteriemia [60,61]. The digestive route would be possible in the case of patients taking drugs that reduce gastric acidity, in this way allowing bacteria to migrate to the colon. A transient bacteriemia could instead be caused by chewing, poor hygiene or dental procedures. Future studies need to shed light on bacterial dissemination in the patient serum.

*Fn* could be considered a biomarker for the staging of CC. This study lays the foundations for future experiments on a larger sample that could detect a colonic cut-off in order to associate its dosage with colic endoscopic investigations for preventive purposes. Oral *Fn* can also be present in adenoma, so it might be interesting to investigate whether oral hygiene sessions could reduce both the oral amount of *Fn* and the risk of colic degeneration. Further research could involve adjuvant treatments for CC against *Fn* invasion, which could affect tumor prognosis in patients with early-stage cancer.

## 4. Material and Methods

### 4.1. Study Cohort

The study population included 36 patients with a histological diagnosis of colon ADK recruited for colonic resection at the Unit of Surgery, Private Hospital “Villa Serena”, Città S. Angelo, Pescara, Italy between July 2018 and August 2019. These individuals presented with altered bowel habits, rectal bleeding or other factors and had a conformed diagnosis of cancer prior to surgery. The study was approved by the Ethics Committee of Chieti on 21 December 2017 (ethic code: RICH1K2HE).

All patients gave written informed consent to participate after oral and written information on the study.

Resected colon cancer and adjacent non-neoplastic tissues from the same patients were taken during surgery, handled with close attention, immediately placed in sterile tubes with 1-mL RNAlater™ (Thermo Fisher Scientific, Waltham, MA, USA) to maintain nucleic acids integrity and stored at −80 °C until DNA extraction.

Exclusion criteria: unstable diabetes, pregnancy or breastfeeding, intake of immunosuppressive drugs, previous chemotherapy and/or radiotherapy within the last 5 years, autoimmune disease (organ transplants or concomitant malignancies), personal history of colon cancer, intestinal diseases related to intestinal dysbiosis, ulcerative colitis and inflammatory bowel disease (IBD). Individuals under antibiotic therapy or using daily Chlorhexidine mouthwash within the last 3 months were also excluded.

In the morning before surgery, 12 h after the last teeth brushing, tongue biofilm was taken from each patient by the same dentist (P.P.) under the same conditions. It consisted of 5 times brushing from the middle-third of the tongue dorsum. After shaking it vigorously for 30 s, the swab was immediately transferred into 5.0 mL of phosphate-buffered saline (PBS) and then kept at +4 °C until the nucleic acid extraction.

Three questionnaires were administered to each patient.

During the oral examination, teeth number, presence/absence of total/partial mobile or fixed prosthesis, the plaque index (PII) and gingival index (GI) of 20 out of 36 patients were recorded in the Oral Health Questionnaire. Briefly, the PII and GI were measured at six surfaces (buccal-mesial, mid-buccal, buccal-distal, lingual-mesial, mid-lingual and lingual-distal) on the Ramfjord teeth (the maxillary right first molar, maxillary left central incisor, maxillary left first premolar, mandibular left first molar, mandibular right central incisor and mandibular right first premolar) with a manual probe (PCP-UNC 15, Hu-Friedy, Chicago, IL, USA).

At enrollment, all patients also completed two self-administered questionnaires: (a) Lifestyle Questionnaire, which included questions about age, sex, body mass index (BMI), smoking status (sig/die) and colon cancer familiarity, and (b) Food Questionnaire, which recorded red and/or processed meat, fruit and vegetable intake, cooking methods and alcohol consumption (unit/die). Alcohol consumption was calculated by converting each frequency of use response into equivalent drinks per week value. Total alcohol consumption per week was calculated as the sum of all individual alcoholic beverage drinks per week values.

Total meat and vegetable intakes were assessed by means of the quantity of consumption (g) for frequency (week) and total fruit intake by means of quantity (g)/frequency (day).

Intakes of fruits, vegetables, meats and alcohol were clustered according to the Passi guidelines (Progress of Health Companies in Italy) [24,54].

### 4.2. Analysis of Oral Bacterial Strains and Growth Conditions

The bacterial strains used in this study were *Fusobacterium nucleatum* (*Fn*) ATCC 25586 and *Porphyromonas gingivalis* (*Pg*) ATCC 33277 (LGC Standards S.r.l., Sesto San Giovanni, Milano, Italy). The strains, stored at −80 °C in a glycerol stock, were thawed at room temperature, plated rapidly on Fastidious Anaerobe Agar (FAA) (Lab M, Heywood, UK) plus 5% of defibrinated horse sterile blood (Oxoid Limited, Hampshire, UK) and incubated at 37 °C for 48 h in an anaerobic atmosphere (Anaerogen Pak Jar, Oxoid Ltd.). After 48 h of incubation, the colonies of *Fn* and *Pg* were washed twice in sterile 0.01-M PBS and centrifuged at 4000× *g* to obtain a visible pellet that was used for the extraction of DNA. Colony-forming unit (CFU) enumeration was carried out by resuspending the samples in 1 mL of PBS; subsequently, serial dilutions of the stock were performed in PBS and plated on FAA. The plates were then incubated at 37 °C for 48 h in an anaerobic atmosphere. Bacterial DNAs were isolated from 4.7 × 10^12^ CFU/mL for *Fn* and 6 × 10^12^ CFU/mL for *Pg* by using a Quick DNA miniPrep Plus kit (Zymo Research, Irvine, USA), obtaining a DNA concentration of 63 ng/μL and 59 ng/μL, respectively.

### 4.3. DNA Extraction

Total genomic DNAs from samples (brushing of the tongue and colon tissues) were extracted by using a Quick DNA miniPrep Plus kit (Zymo Research), suitable for DNA extraction from both tissue and buccal swab with brushing, according to the manufacturer’s instructions, with minor modifications.

DNA was isolated from about 25mg of frozen colon tissues homogenized using a mortar and pestle with liquid nitrogen using the TRIzol^®^ reagent (Applied Biosystems, Thermo Fisher Scientific, Waltham, MA, USA), according to the manufacturer’s instructions.

Cells from brushing were pelleted by centrifugation at 4000 rpm for 5 min, and then, pellets were dissolved in 700 μL of TRIzol^®^ Reagent.

Then, 200 μL of chloroform were added to both suspensions (oral cells and colon/tissue cells). After mixing them vigorously and incubating them for 2 to 3 min, samples were centrifugated at 13,000 rpm for 15 min at 4 °C. The DNA, collected from the interfaces, was precipitated by mixing with isopropanol and with cold ethanol with several washes.

Subsequent steps were performed according to Zymo Research’s recommendations. The protocol included a 30-min treatment with 10-μL proteinase K (20 mg/mL) at 70 °C. After isolation and purification, DNA was eluted in 60 μL of elution buffer. DNA was quantified on a NanoDrop Spectrophotometer (Thermo Fisher Scientific), and DNA concentration ranged from 12 ng/μL to 32 ng/μL.

### 4.4. Bacterial DNA Quantification by qPCR Analysis

qPCR analysis using StepOne™ 2.0 (Applied Biosystems, Thermo Fisher Scientific, Waltham, MA, USA) was performed to quantify the abundance of *Fn* and *Pg* in our samples.

Each reaction contained 1 μL of total DNA and was assayed in triplicate in 10-μL qPCR reactions containing 5 μL of 2× Universal Master Mix, as previously reported [62].

For *Fn*, a Syber green-based assay was used to quantify the FadA gene using the following primers: 5′- CAAATCAAGAAGAAGCAAGATTCAAT-3′ forward primer and 5′-GCTTGAAGTCTTTGAGCTCT-3′ reverse primer [25,63].

For *Pg*, a TaqMan-based assay was performed, according to Uraz et al. [64], to quantify *Pg* 16S rRNA, the gene encoding the small subunit of 16S ribosomal RNA. The TaqMan Gene Expression Custom Assay was provided by Life Technologies (Thermo Fisher Scientific) with the following primers: 5′-GCGCTCAACGTTCAGCC-3′ forward primer, 5′-CACGAATTCCGCCTGC-3′ reverse primer and probe FAM-CACTGAACTCAAGCCCGGCAGTTTCAA-TAMRA.

We selected the standard curve method as the quantitation method to determine the target quantity in the samples. Briefly, according to the manufacturer’s instructions, five serial 10-fold dilutions of the corresponding bacterial DNA used as the standard were prepared, ranging from 4.7 × 10^11^ to 4.7 × 10^7^ CFU/mL for *Fn* and from 6 × 10^10^ to 6 × 10^6^ CFU/mL for *Pg* (Supplementary Figure S2). Based on this standard curve passing through the 5 points, indicating the cycle threshold value versus *Fn* FadA gene and *Pg* 16S rRNA gene, respectively, it was possible to estimate the bacterial quantity related to the amount of total DNA isolated from the oral and tissue samples used in the qPCR reaction. For those values apparently distant from the others, an additional qPCR using the same samples and methods was performed, obtaining almost identical values.

### 4.5. Data Analysis

The statistical analyses were carried out using the R-based open-source software Jamovi Version 1.2.5.0 (retrieved from https://www.jamovi.org). A Shapiro-Wilk test was performed on all variables to check the normality of distribution. Variables that did not pass the normality test were log-transformed. Consequently, GI, *Fn* and *Pg* brushing quantity; *Fn* quantity in tumoral tissue and *Fn* quantity in adjacent tissue underwent a log_10_ transformation.

A series of general linear models with Tukey’s post-hoc correction was performed to test separately each oral health index or microbial quantity predicted by lifestyle or dietary habit variables. In particular, the predictors were the fruit, vegetable, or meat consumptions; smoking habit or alcoholic consumption, and the response variables were the number of teeth, gingival index, plaque index or quantity of microbial populations. Levene’s test for the homogeneity of residual variances and Kolmogorov-Smirnov test for normality of the residuals were performed. Concerning the number of teeth, two patients with no teeth were removed for this analysis; partial eta squared (η^2^*_p_*) and adjusted R^2^ (R^2^_adj_) were calculated. ANOVA was used to compare the *Fn* quantity in diverse cancer localizations (ascending vs. transverse vs. descending vs. sigmoid colon). Levene’s tests for homoscedasticity was used as assumption check. Welch’s test was also used to compare the *Fn* and *Pg* quantities in men vs. women. Both robust paired-sample *t*-test (10 percent trimmed mean) and Wilcoxon signed-rank test were used to compare *Fn* in brushing, tumor tissue and *Fn* in adjacent non-neoplastic tissue. The effect size measure Cohen’s d was adjusted to Cohen’s dunbiased [41].

The software Gephi 0.9.2 [65] was used for the network analysis. This analysis allowed to plot the relationships between age, BMI, oral health indices and microbial quantities in cancer patients as the nine nodes of an undirected weighted network. Weights were represented by nonparametric Spearman correlation coefficients (rho). Nodes were positioned using the Fruchterman-Reingold algorithm. The threshold of 0.24, which corresponds to a Cohen’s d value of 0.5 [41], was used as the minimum weight to plot the edges between nodes. Eigenvector [43], betweenness and closeness [44] were calculated as the measures of centrality.

A series of linear regressions were conducted to test the role of *Fn* in oral tissue in predicting *Fn* in healthy gut tissue and *Fn* in tumoral gut tissue. Durbin-Watson test for autocorrelation [66], Shapiro-Wilk test for normality and Q-Q plots observation were used as assumption checks. The model fit R and root mean square error (RMSE) measures were calculated. A series of ordinal logistic regressions were conducted to test separately the role of each microbial population in predicting the cancer staging or grading, calculating McFadden’s R2, Nagelkerke’s R2 and the odds ratio. Consequently, a series of mediation analyses were conducted to estimate the percentage of mediation exerted by age or BMI on the conditional dependence between (1) *Fn* in oral and tumoral tissue and (2) *Fn* in tumoral tissue and cancer staging.

The achieved statistical power (1−β) and the required sample size for further analyses were computed with the software G*Power Version 3.1.9.3.

## 5. Conclusions

In our study, we showed a direct relationship between *Fn* abundance in pathological tissue and increased severity of colon cancer. It was evident that an increase in oral *Fn* concentrations was correlated with an increase in colorectal tissue *Fn* quantity. This study also revealed a tendency for high-meat consumers to have greater *Fn* quantities both in adjacent non-neoplastic tissue and in cancer tissue. *Pg* was not detected in colon tissues but was correlated with the gingival and plaque indices, confirming its important role in oral inflammation and a noninvolvement of this bacteria in colon cancer. *Fn* could be considered a biomarker for the staging of CC. Further studies on a greater sample size would be required to see if alternative periodontal indices may correlate with the concentration of oral *Fn* and to detect a colonic cut-off in order to associate the *Fn* dosage with colic endoscopic investigations for preventive purposes.

## Figures and Tables

**Table 1:** Clinical and histopathological characteristics of patients with colon ADK.

Case	Age at Diagnosis	Sex	Site of Tumor	T	N	M	Grading	Staging	Comorbidity
1C	73	F	R	3	0	X	3	2	-
2C	67	M	L	3	0	X	2	2	DM
3C	67	F	L	2	0	X	2	2	-
4C	55	F	R	3	1b	X	2	3	MS
5C	84	M	R	3	0	X	2	2	AH
6C	30	F	SIG	2	2	X	2	3b	-
7C	82	M	R	3	2b	X	2	3c	AH
8C	47	M	R	3	1	X	3	3	-
9C	73	F	R	2	0	X	INDIFF	2	AH, IE, PMK, DM
10C	71	-	SIG	3	0	X	-	2	-
11C	79	M	R	1	0	X	3	1	-
12C	71	F	SIG	1	0	X	2	1	-
13C	61	F	L	3	1b	X	2	3	DM, asthma
14C	52	M	SIG	4	1b	X	2	3b	-
15C	68	M	R	2	0	X	3	1	-
16C	64	M	SIG	4b	1b	X	MUC	3b	AH
17C	77	F	SIG	2	0	X	2	1	-
18C	71	M	R, L	4b	2a	1	2	4a	-
19C	87	F	R	2	0	X	2	1	-
20C	64	M	R	3	0	X	3	2a	-
21C	66	M	SIG	2	0	X	2	1	-
22C	81	F	R	2	0	X	3	1	SLC
23C	65	F	SIG	2	0	X	1	1	-
24C	66	F	SIG	3	1c (td)	X	2	3b	OCA L
25C	75	F	R	3	1	X	2	3b	-
26C	74	M	TR	3	0	X	2	2a	-
27C	60	F	TR	3	N1c	1	2	4a	LM
28C	67	F	R	T4bv	2b	X	2	3c	-
29C	46	M	SIG	IN SITU	0	X	1	0	-
30C	75	F	SIG	3v1	N1c	X	2	3b	-
31C	73	M	R	3	0	X	3	2a	-
32C	40	F	TR	3v1	0	1	3	4a	SLM
33C	76	F	R	2	0	X	2	1	-
34C	80	M	L	2v1	0	X	2	1	-
35C	61	M	R	3	0	X	MUC	2a	S
36C	70	M	n.a.	n.a.	n.a.	n.a.	n.a.	n.a.	-

AH: Arterial Hypertension, DM: Diabetes Mellitus, IE: Infective Endocarditis, L: Left, LM: Liver Metastases, MS: Multiple Sclerosis, MUC: Mucinous, OCA: Ovarian Cystadenoma, OPMK: Pacemaker, S: Synchronous, SLC: Synchronous Lung Cancer, SLM: Synchronous Liver Metastases, R: Right, UND: Undifferentiated, n.a.: Not Applicable and ADK: Adenocarcinoma.

**Table 2:** Demographic and lifestyle characteristics of the study subjects.

Characteristics	Categories	Disease History Colon Cancer *N*	%	Mean ± SD
Smoking	≤10 cigs/day	3	9.4	14.38 ± 4.17
	>10 cigs/day	5	15.6	
	former and not	24	75	
Alcohol	≤15 g/day F ≤30 g/day M	28	87.5	12.69 ± 11.43
	>15 g/day F >30 g/day M	4	12.5	
Body mass index (kg/m^2^)	<25	18	60	25.05 ± 4.21
	25–29.9	9	30	
	≥30	3	10	
GI	-	20	-	0.66 ± 0.67
PII	-	20	-	1.4 ± 0.73
N. teeth	-	20	-	21.05 ± 9.10

Seventy-five% of the sample was nonsmoking, 87.5% light drinkers and 60% had healthy weights. Most of the colon cancer patients did not have bad life habits but did not have optimal oral hygiene. CIGS: cigarettes, GI: gingival index and PII: plaque index.

**Table 3:** Correlation matrices of the oral health parameters and *Fn* and *Pg* abundance. Rho coefficients were calculated with Spearman’s method.

Variable	BMI	PlI	GI	*Fn* Oral	Fn T	*Fn* Adj T	*Pg* Oral	Age
PlI	−0.22	-	-	-	-	-	-	-
GI	0.05	0.52 *	-	-	-	-	-	-
*Fn* oral	0.29	−0.14	0.15	-	-	-	-	-
*Fn* T	−0.12	−0.23	0.12	0.26	-	-	-	-
*Fn* adj t	0.10	−0.22	0.12	0.38 *	0.80 ***	-	-	-
*Pg* oral	−0.09	0.23	0.38	−0.01	0.09	0.24	-	-
Age	0.35	−0.05	−0.16	−0.21	−0.23	−0.15	−0.23	-
N. teeth	0.05	0.14	−0.33	−0.15	−0.20	0.05	0.36	0.11

Adj t: Adjacent non-neoplastic tissue, BMI: body mass index, *Fn*: Fusobacterium nucleatum, GI: gingival index, N.: number, PlI: plaque index, *Pg*: Porphyromonas gingivalis and T: Tumor tissue. Note: * *p* < 0.05 and *** *p* < 0.001.

**Table 4:** Statistics of the ordinal logistic regression. *Fn* quantities were defined as predictors and staging or grading as ordinal variables. McFadden’s and Nagelkerke’s R^2^ (_McF_R^2^ and _N_R^2^, respectively) were chosen as the effect size measures. Of note was that the lesser are Akaike information criterion (AIC) and Bayesian information criterion (BIC), and the better is the fit of the model.

*Fn* Location	Variable	*p*-Value	_McF_R^2^	_N_R^2^	Odds Ratio	AIC	BIC
*Fn* oral	Staging	0.507	0.006	0.008	1.205	86.17	91.77
	Grading	0.225	0.028	0.034	1.539	59.93	65.40
*Fn* T	Staging	0.016	0.077	0.102	1.771	78.50	83.97
	Grading	0.296	0.020	0.025	1.294	61.14	66.47
*Fn* adj t	Staging	0.088	0.045	0.061	1.914	69.45	74.34
	Grading	0.199	0.034	0.043	1.592	54.99	56.70

adj t: Adjacent non-neoplastic tissue and T: Tumor tissue.

## Data Availability

The datasets used and/or analyzed during the current study are available from the corresponding author on reasonable request.

## Supplementary Materials

The following are available online at https://www.mdpi.com/2072-6694/13/5/1032/s1: Figure S1: The figure shows the amounts of dietary meat, vegetables and fruits, divided into high and low, Figure S2: The figure shows a representative example of a standard curve obtained by real-time PCR to quantify the bacterial DNA (*Fn* and *Pg)* in the samples, Table S1: *Fn* and *Pg* quantities in the oral cavity and matched adjacent non-neoplastic mucosa (adj t) and cancer tissue (T) by qPCR, Table S2: Statistical analysis of the comparison between Fn in brushing, tumor tissue and adjacent non-neoplastic tissue.

## Abbreviations

Colorectal cancer	CRC
Colon Cancer	CC
Adenocarcinoma	ADK
*Fusobacterium nucleatum*	*Fn*
*Porphyromonas gingivalis*	*Pg*
adjacent non-neoplastic mucosa	adj t
vascular endothelial cadherin	VE-cadherin
mucosa-associated lymphoid tissue	MALT
T-lymphocyte-infiltrating tumor	TIL
reactive oxygen species	ROS
Plaque index	PII
Gingival index	GI
Body mass index	BMI
Root mean square error	RMSE
Colony-Forming Unit	CFU
ultra-processed foods	UPFs
quantitative real-time PCR	qPCR
polymicrobial synergy and dysbiosis	PSD
