# Supplementary material for: The Potential of Colonic Tumor Tissue Fusobacterium nucleatum to Predict Staging and Its Interplay with Oral Abundance in Colon Cancer Patients
Source: Cancers (Basel). 2021 Mar 1;13(5):1032. doi: 10.3390/cancers13051032 (PMC7957509; doi:10.3390/cancers13051032)
Supplement: Supplementary file 1 [file cancers-13-01032-s001.pdf]

Supplementary Materials: The Potential of Colonic Tumor Tissue *Fusobacterium Nucleatum* to Predict Staging and Its Interplay with Oral Abundance in Colon Cancer Patients

Pamela Pignatelli, Lorena Iezzi, Martina Pennese, Paolo Raimondi, Anna Cichella, Danilo Bondi, Rossella Grande, Roberto Cotellese, Nicola Di Bartolomeo, Paolo Innocenti, Adriano Piattelli and Maria Cristina Curia

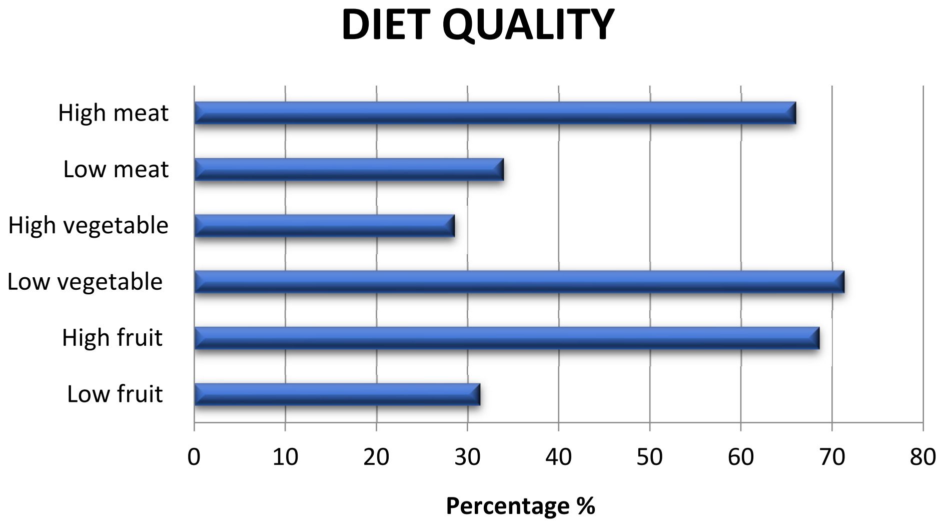

Figure S1. The figure shows the amounts of dietary meat, vegetable and fruit**,** divided into high and low. Most of the sample had a diet rich in meat (66% of the patients), fruit (68.6% of the patients) and poor in vegetables (71.4% of the patients). Low fruit intake: <150 g/die; High fruit intake: 150–450 g/die; Low vegetable intake: <200 g/die; High vegetable intake: 200–400 g/die; Low meat intake: 0–400 g/we; High meat intake: 400–600 g/we.

Table S1. *Fn* and *Pg* quantities in the oral cavity and matched adjacent non-neoplastic mucosa (adj t) and cancer tissue (T) by qPCR.

Case	*Fusobacterium nucleatum*						*Porphyromonas gingivalis*	
	Oral (Med = 108.69, IQR = 300.63)		adj t (Med = 2.19, IQR = 8.96)		T (Med = 4.78, IQR = 68.20)		Oral (Med = 0.34, IQR = 38.88)	
	CFU/mL	SD	CFU/mL	SD	CFU/mL	SD	CFU/mL	SD
1C	0	0	0.34	0.05	0.58	0.06	0	0
2C	0.21	0.02	0	0	0.01	0	2209.92	1.77
3C	208.92	9.89	23.36	0.92	54.59	1.68	0.48	0.045
4C	157.98	4.34	11.42	0.79	202.81	12.95	196.46	18.64
5C	2.38	0.10	0	0	0.01	0	0	0
6C	12.01	0.05	22.53	0.42	604.84	46.39	6.96	1.42
7C	51.46	5.13	220.84	7.72	207.11	15.40	0.07	0.04
8C	76.54	10.97	5.02	0.06	63.24	4.89	0	0
9C	133.92	7.64	7.97	0.18	4.31	0.15	0	0
10C	3083.20	426.06	0	0	26.21	1.14	0	0
11C	38.82	0.12	0.48	0.02	0.66	0.05	0	0
12C	105.81	11.23	3.42	0.07	2.01	0.03	64.11	6.93
13C	111.56	3.64	3.74	0	159.82	0.31	2.63	0.25
14C	177.15	27.27	0	0	0	0	0	0
15C	1.55	0.46	1.56	0.44	0.55	0.02	0.19	0.01
16C	302.55	18.29	2.19	0.32	94.75	110.63	0	0
17C	48.45	0.44	0.23	0.09	0.32	0.08	0.12	0
18C	307.52	4.99	40.68	1.07	307.52	4.99	1.00	0
19C	132.92	1.85	1.21	0.24	85.41	30.13	0.05	0.01
20C	525.98	53.76	3.49	0.93	3.54	1.43	1961.24	401.80
21C	19.79	0.21	28.08	0.38	3.91	0.42	234.68	2.92
22C	1800.40	83.60	3.68	0.70	9.00	1.35	0	0
23C	1.20	0.07	1.16	0.31	0.03	0	2.38	0.50
24C	2.42	0.19	8.63	0.32	45.29	3.09	1.15	0.03
25C	0.33	0.02	0.06	0.01	0.27	0.05	41.14	1.10
26C	8421.99	774.58	18.43	2.01	7.22	1.67	0	0
27C	33.86	1.29	0	0	5.82	0.21	0	0
28C	0	0	0	0	0	0	655.00	47.93
29C	1724.62	34.79	0	0	0.01	0	-	-
30C	0	0	0	0	23.53	0.70	269.36	27.64
31C	172.13	12.82	1551.87	26.46	5833.85	249.62	32.10	5.79
32C	2156.26	51.12	10.10	1.23	0.12	0.01	-	-
33C	130	3.34	0	0	0	6.05	-	-
34C	3.41	0.27	0.05	0	0.02	0.01	-	-
35C	2210.54	276.64	2.18	0.03	84.02	2.53	-	-
36C	1781.30	15.69	6.18	0.55	5.25	0.69	-	-

The numerical values (CFU) represent the mean of triplicate determinations with standard deviation (SD). The concentration of the PCR products was converted to CFU using five serial dilutions (1:10) employed to create the standard curve (ranging from 4.7×10^{11} CFU/mL = 6.3 ng/µl to 4.7×10^{7} CFU/mL = 6.3×10^{-4} ng/µl for *Fn*; from 6×10^{10} CFU/mL = 5.9×10^{-1} ng/µl to 6×10^{6} = 5.9×10^{-5} ng/µl for *Pg*). Med: median; IQR: interquartile range.

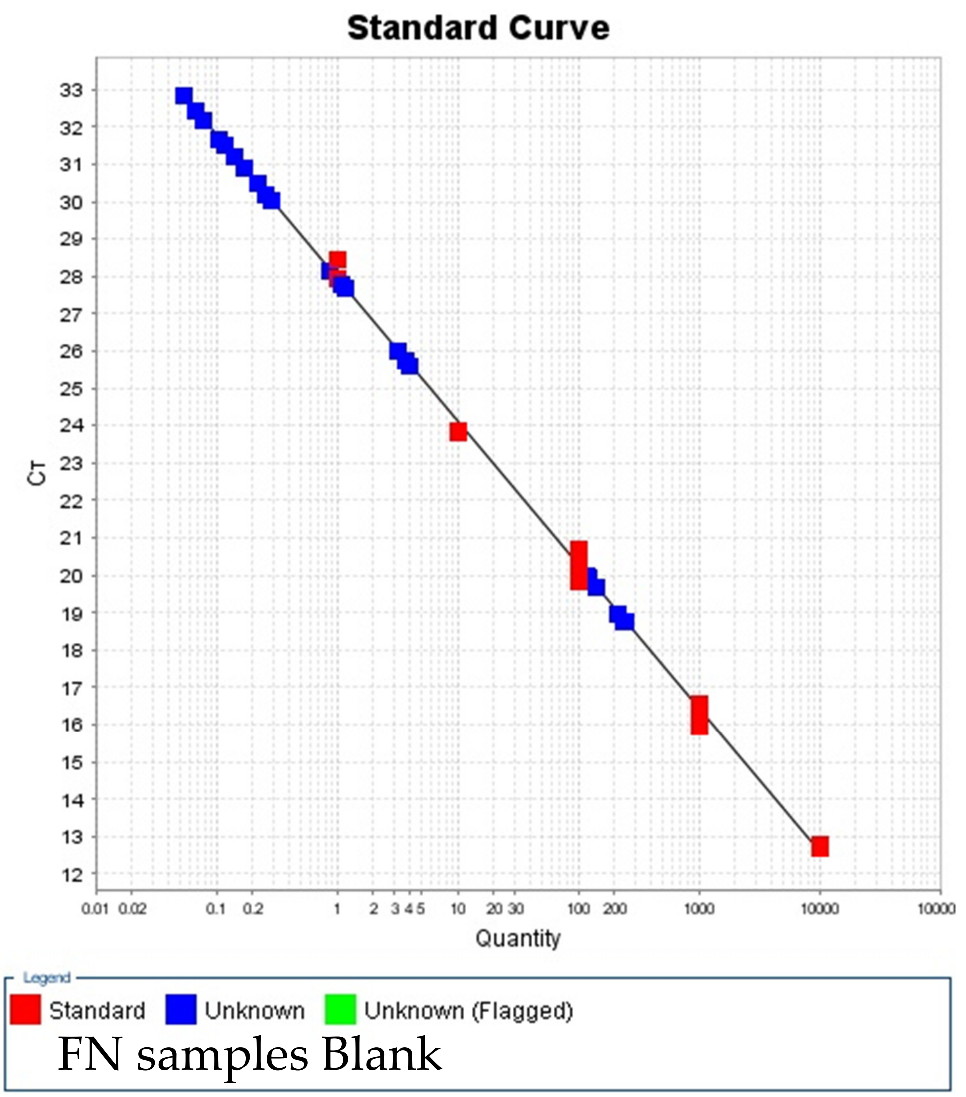

Figure S2. The figure shows a representative example of a standard curve obtained by Real-Time PCR to quantize bacterial DNA (*Fn* and *Pg)* in the samples.

Red squares: five *Fn* ATCC 25586 serial dilutions used as standard.
Quantity CFU/mL [DNA ng/µl]

1. $10000 = 4.7 \times 10^{11} = 6.3$
2. $1000 = 4.7 \times 10^{10} = 6.3 \times 10^{-1}$
3. $100 = 4.7 \times 10^{9} = 6.3 \times 10^{-2}$
4. $10 = 4.7 \times 10^{8} = 6.3 \times 10^{-3}$
5. $1 = 4.7 \times 10^{7} = 6.3 \times 10^{-4}$

For *Pg*: five ATCC 33277 serial dilutions used as standard.
Quantity CFU/mL [DNA ng/µl]

1. $6 \times 10^{10} = 5.9 \times 10^{-1}$
2. $6 \times 10^{9} = 5.9 \times 10^{-2}$
3. $6 \times 10^{8} = 5.9 \times 10^{-3}$
4. $6 \times 10^{7} = 5.9 \times 10^{-4}$
5. $6 \times 10^{6} = 5.9 \times 10^{-5}$

Table S2. Statistical analysis of the comparison between *Fn* in brushing, tumor tissue, and adjacent non-neoplastic tissue.

Fn	Wilcoxon Test	Robust Test	Effect Size
Brushing vs. tumor tissue	$p = 0.004$	$p = 0.059$	$d_{unb} = 0.332$
Brushing vs. adjacent non-neoplastic tissue	$p < 0.001$	$p = 0.038$	$d_{unb} = 0.382$
Tumor tissue vs. adjacent non-neoplastic tissue	$p = 0.100$	$p = 0.066$	$d_{unb} = 0.253$

Wilcoxon signed-rank test and robust paired sample *t*-test were used because of the non-normal distributions of data.

Copyright: © 2021 by the author. Licensee MDPI, Basel, Switzerland. This article is an open access article distributed under the terms and conditions of the Creative Commons Attribution (CC BY) license (http://creativecommons.org/licenses/by/4.0/).
